# Twelve-month smoking cessation outcomes following immediate referral in people who smoke with chronic airway diseases: a randomized study

**DOI:** 10.1186/s13722-026-00676-0

**Published:** 2026-05-12

**Authors:** Dilek Karadoğan, Tahsin Gökhan Telatar, İlknur Kaya, Siahmet Atlı, Neslihan Köse Kabil, Feride Marım, Merve Yumrukuz Şenel, Aycan Yüksel, Burcu Yalçın, Ökkeş Gültekin, Merve Erçelik, Metin Akgün

**Affiliations:** 1 Department of Chest Diseases, School of Medicine, Recep Tayyip Erdoğan University, Rize, Türkiye; 2 Department of Public Health, School of Medicine, Recep Tayyip Erdoğan University, Rize, Türkiye; 3 Department of Chest Diseases, Faculty of Medicine, Kutahya Health Sciences University, Kütahya, Türkiye; 4 Department of Chest Diseases, Faculty of Medicine, Fırat University, Elazığ, Türkiye; 5 Department of Chest Diseases, Yalova University, Yalova, Türkiye; 6 Department of Chest Diseases, Faculty of Medicine, Balikesir University, Balıkesir, Türkiye; 7 Department of Chest Diseases, Faculty of Medicine, TOBB Economy and Technology University, Ankara, Türkiye; 8 Department of Chest Diseases, Merzifon Karamustafapasa State Hospital, Amasya, Türkiye; 9 Department of Chest Diseases, Kemalpaşa State Hospital, İzmir, Türkiye; 10 Department of Chest Diseases, Faculty of Medicine, Süleyman Demirel University, Isparta, Türkiye; 11 Department of Chest Diseases, School of Medicine, Ağrı İbrahim Çeçen University, Ağrı, Türkiye

**Keywords:** Smoking cessation, Asthma, COPD, Immediate appointment, Randomized trial

## Abstract

**Background:**

Tobacco cessation support remains underutilized in routine care for patients with airway diseases such as asthma and COPD. In this multicenter randomized trial (NCT05764343, registration date: 2023-03-01), we previously reported that immediately scheduled appointments to smoking cessation clinics improved access and quit rates at 1-week and 3-month follow-ups. The present study evaluated whether these effects were sustained at 12 months.

**Methods:**

This prospective, parallel-arm, multicenter randomized trial included 397 adult people who smoke diagnosed with asthma, COPD, or bronchiectasis. Participants were allocated to either usual support (brief advice only) or immediate support (brief advice plus an appointment scheduled at a smoking cessation clinic). Smoking status was assessed by telephone at 12 months. Self-reported quitters were invited for exhaled carbon monoxide (CO) testing. The primary outcome was continuous abstinence at 12 months, analyzed on an intention-to-treat basis.

**Results:**

Of 397 randomized patients, 330 (83.1%) completed the 12-month follow-up, with similar loss to follow-up between groups. In the intention-to-treat analysis, the 12-month smoking cessation rate was significantly higher in the immediate support group compared with the usual support group (20.7% vs. 11.6%, *p* = 0.019). Among non-quitters, quit attempts, smoking cessation clinic admission, and pharmacotherapy use were significantly more common in the immediate support group (*p* < 0.05).

**Conclusion:**

Immediate scheduling of smoking cessation clinic appointments resulted in significantly higher 12-month quit rates compared to usual care. These findings support the integration of proactive referral strategies into routine management of patients with chronic airway diseases.

## Introduction

Chronic airway diseases such as asthma and chronic obstructive pulmonary disease (COPD) remain leading causes of global morbidity and mortality. Tobacco smoking is the principal modifiable risk factor for COPD and a major contributor to disease progression, increased exacerbation frequency and severity, and reduced quality of life in both conditions [1, 2]. Smoking cessation is the most effective and cost-efficient intervention for improving clinical outcomes, preserving lung function, and reducing healthcare utilization in both asthma and COPD [3, 4].

Despite the well-documented benefits of cessation, smoking remains alarmingly prevalent among individuals with chronic airway diseases. Studies report that around 40% of patients with COPD and up to 20% of those with asthma continue to smoke even after diagnosis [5, 6]. Persistent tobacco use in these populations has been linked to higher nicotine dependence, frequent psychiatric comorbidities, lower health literacy, and various psychosocial and environmental stressors [7, 8]. Furthermore, access to evidence-based cessation resources—such as pharmacotherapy and counseling—is often delayed or absent in routine care. A multicenter observational study conducted across pulmonology clinics in Türkiye found that only 1.9% of current smokers with COPD had accessed a smoking cessation clinic, and the vast majority of both asthma and COPD patients had not utilized national quitlines, highlighting a critical treatment gap in real-world settings [9].

To address this gap, a two-phase multicenter randomized controlled trial was conducted (NCT05764343) to test the effect of proactively scheduling smoking cessation clinic (SCC) appointments for patients with chronic airway diseases. In the first study, which evaluated outcomes at 1-week, patients who received an immediate SCC appointment were significantly more likely to attend the clinic within one week (77.3% vs. 18.1%, *p* < 0.001) [10]. At 3rd months follow ups, intervention arm was more likely to attempt quitting, and demonstrated a significantly higher 3-month abstinence rate compared to usual care (26.7% vs. 16.5%, *p* = 0.014) [11]. Access to pharmacological cessation treatments was markedly better in the intervention arm (69.3% vs. 22.0%), and multivariate analysis identified pharmacotherapy access as the strongest independent predictor of early quit success [11].

However, the durability of these early gains over longer follow-up remained unclear. Given that relapse is common within the first year of quitting, particularly in populations with chronic disease, a 12-month follow-up assessment was conducted within the original randomized controlled trial cohort to evaluate long-term outcomes. Although this extended follow-up was not pre-specified in the initial study design, it enabled assessment of whether the initial benefits of immediate SCC referral—specifically, enhanced access, engagement, and early quit success—were sustained as long-term abstinence one year after the intervention.

## Methods

This study presents the 12-month follow-up results of a previously approved prospective, multicenter, randomized controlled trial (RCT) conducted between November 2022 and June 2023 (ClinicalTrials.gov Identifier: NCT05764343, registration date: 2023-03-01). Although the 12-month follow-up was not pre-specified in the original study protocol, participants from the original RCT cohort were reassessed to evaluate the long-term sustainability of the intervention effects. The study protocol was approved by the RTEU Clinical Research Ethics Committee (Approval No. 2022/09, Date: 08.11.2022), with additional approval for the extended 12-month follow-up obtained on May 2, 2024 (Approval No. 2024/95). The study was conducted in accordance with the Declaration of Helsinki, and written informed consent was obtained from all participants. All 12-month follow-up calls were completed between May 2, 2024, and June 15, 2024, ensuring a uniform assessment window for the cohort.

A total of 397 adult patients who were current smokers with a confirmed diagnosis of chronic airway disease—specifically asthma, COPD, or bronchiectasis—were recruited across 11 pulmonary outpatient clinics in Türkiye. All patients were randomized in a 1:1 ratio to receive either usual support or immediate support. The usual support group received routine care, consisting of brief cessation counseling and encouragement to independently access cessation services such as national quitlines or outpatient clinics. In contrast, the immediate support group received the same brief intervention plus an appointment scheduled promptly at a smoking cessation outpatient clinic within the same institution.

Participants were allocated using an alternating sequence (e.g. the first patient to one group, the second to the other), rather than computer-generated randomization. Allocation concealment was not used. Due to the nature of the intervention, blinding of participants and clinical staff was not feasible. However, outcome assessment was conducted by trained researchers who were blinded to group assignments.

Twelve months after randomization, all participants were contacted by telephone. Follow-up interviews were conducted using a standardized format to assess smoking status, including whether the participant had quit, relapsed, or was still smoking. Additional information was collected regarding any smoking cessation support used in the past year, including attendance at cessation clinics, type and duration of pharmacologic treatments (e.g. nicotine replacement therapy, bupropion, or varenicline), and any respiratory events such as disease exacerbations or hospitalizations related to lung disease. Smoking status was based on self-report. Participants who declared continuous abstinence since their target quit date were invited to attend the clinic for biochemical verification using exhaled carbon monoxide (CO) testing; a CO level below 7 ppm was accepted as confirmation of abstinence.

The primary outcome was continuous smoking abstinence at 12 months, evaluated using the intention-to-treat principle. All randomized patients were included in the denominator, with those lost to follow-up assumed to be people who smoke. Secondary outcomes included quit attempts (including those who relapsed), utilization of pharmacotherapy, and healthcare utilization related to respiratory disease.

### Statistical analysis

All statistical analyses were conducted using IBM SPSS Statistics version 29 (IBM Corp., Armonk, NY) and R 4.5.2 (R Foundation for Statistical Computing, Vienna, Austria). Descriptive statistics were used to summarize baseline characteristics and study outcomes. Categorical variables were expressed as counts and percentages, while continuous variables were presented as mean ± standard deviation (SD) or median (interquartile range, IQR), depending on distribution normality. Normality of continuous variables was evaluated using the Kolmogorov–Smirnov test and visual inspection of histograms.

The primary outcome—12-month continuous abstinence—was analyzed according to the intention-to-treat (ITT) principle, with all randomized participants included in the denominator and those lost to follow-up classified as people who smoke. Group comparisons for categorical outcomes (quit rate, quit attempts, pharmacotherapy use, relapse) were performed using Pearson’s chi-square test or Fisher’s exact test when appropriate. For continuous variables such as treatment duration, between-group differences were assessed using the independent samples *t*-test or the Mann–Whitney *U* test based on data distribution.

All statistical tests were two-tailed, and a *p*-value < 0.05 was considered statistically significant.

## Results

**Fig. 1:**
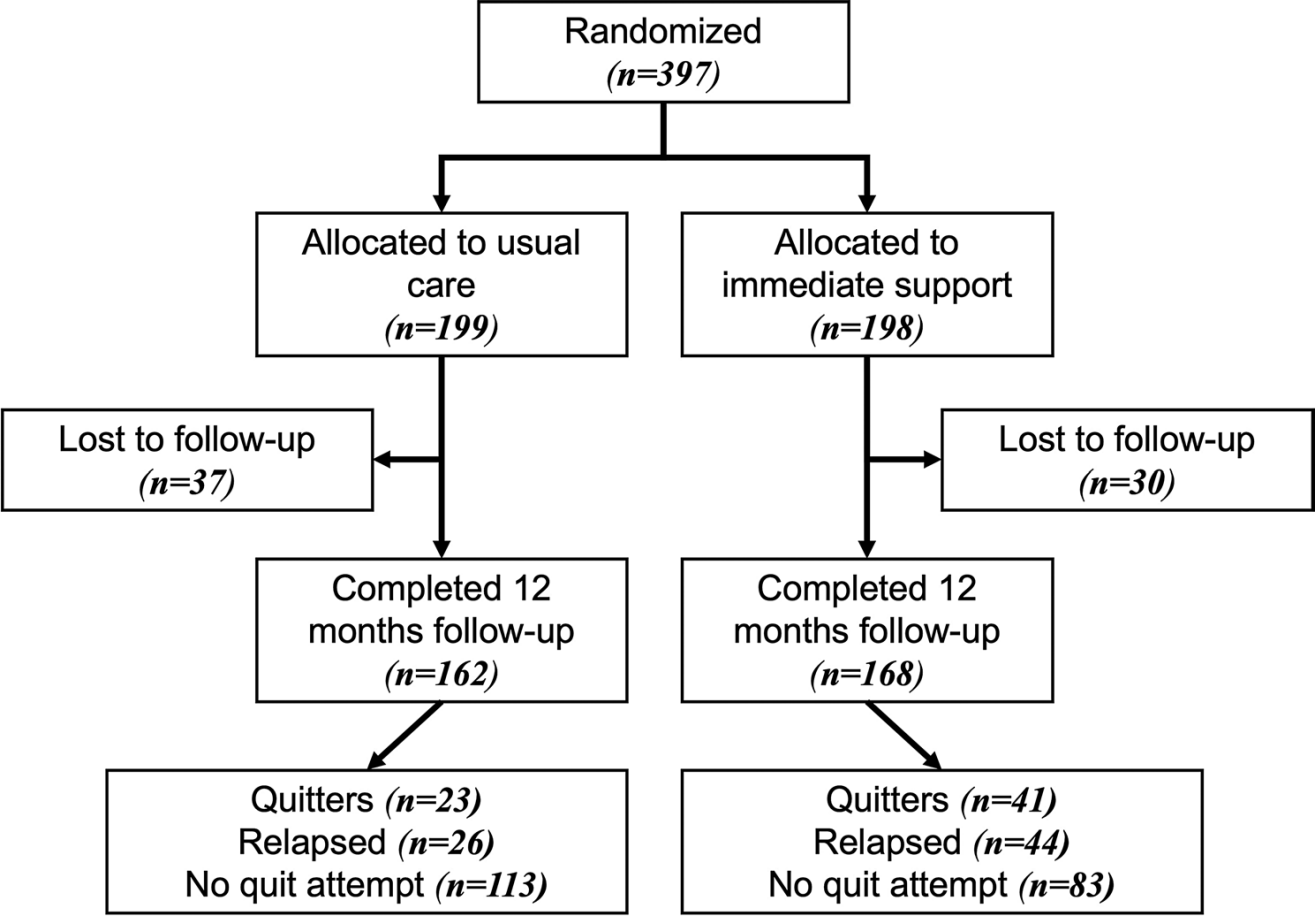
CONSORT flow diagram of participant enrollment, allocation, follow-up, and analysis

Of the 397 patients randomized, 330 (83.1%) completed the 12-month follow-up. The usual support group comprised 162 patients, while the immediate support group included 168 (Fig. 1). Loss to follow-up occurred in 17% of participants and was similar across both arms. A total of 67 participants (16.9%) were lost to follow-up, corresponding to 18.6% in the usual care arm and 15.2% in the intervention arm. A comparison of baseline characteristics between participants lost to follow-up and those who completed the study is presented in Table 1. Patients lost to follow-up were older (mean age 56.1 vs. 53.1 years) and had higher baseline nicotine dependence (mean Fagerström score 7.31 vs. 5.93). There was a significant difference in diagnosis distribution between the groups (*p* = 0.008), with COPD being more prevalent among those lost to follow-up (71.6% vs. 52.1%), whereas asthma was less frequent (28.4% vs. 44.2%). The proportion of bronchiectasis patients was low in both groups.

**Table 1:** Baseline characteristics of participants according to follow-up status (completed vs. lost to follow-up)

Lost to follow-up	*n*	Mean age	Mean score for FTND	COPD %	Asthma %	Bronchiectasis %
No	330	53.1	5.93	52.1	44.2	3.64
Yes	67	56.1	7.31	71.6	28.4	0.0

FTND: Fagerström test for nicotine dependence

At 12-month follow-up (Table 2), sustained quitters were more frequent in the immediate support group than in the usual support group (41/168, 24.4% vs. 23/162, 14.2%; *p* = 0.027). Among sustained quitters, admission to the Smoking Cessation Clinic (SCC) and use of pharmacotherapy were significantly higher in the immediate support group (33/41, 80.5% vs. 8/23, 34.8%; *p* = 0.001; and 32/41, 78.0% vs. 7/23, 30.4%; *p* = 0.001, respectively). Among non-quitters, quit attempts were more common in the immediate support group (44/127, 34.6% vs. 26/139, 18.7%; *p* = 0.005), with higher SCC admission (95/127, 74.8% vs. 35/139, 25.2%; *p* < 0.001) and pharmacotherapy use (94/127, 74.0% vs. 46/139, 33.1%; *p* < 0.001).

**Table 2:** Comparison of quit outcomes and treatment utilization between intervention arms

Category	Usual Support (*n* = 162)	Immediate Support (*n* = 168)	*p*
Sustained Quitters	23 (14.2%)	41 (24.4%)	0.027
Admitted to SCC	8 (34.8%)	33 (80.5%)	0.001
Used Pharmacotherapy	7 (30.4%)	32 (78.0%)	0.001
Support used to quit	Patch (1), Bupropion (4), Other (2)	Patch (17), Bupropion (11), Other (4)	-
Duration of SCM (days)	46.5 (36.3)	39.6 (32.4)	0.014
Non-quitters	139 (85.8%)	127 (75.6%)	-
Relapsed (Quit attempt made)	26 (18.7%)	44 (34.6%)	0.005
No Quit Attempt	113 (81.3%)	83 (65.4%)	-
Admitted to SCC	35 (25.2%)	95 (74.8%)	< 0.001
Used Pharmacotherapy	46 (33.1%)	94 (74.0%)	< 0.001
Support used in quit attempts	Patch (10), Bupropion (19), Other (14)	Patch (29), Bupropion (47), Combo (9), Other (9)	
Duration of SCM (days)	22.7 (16.7)	21.2 (20.5)	0.097

SCC: Smoking Cessation Clinic, SCM: Smoking Cessation Medication, Patch: Nicotine Replacement Therapy Patch

According to the intention-to-treat (ITT) analysis, which included all randomized patients and assumed those lost to follow-up were people who smoke, the smoking cessation rate at 12 months was significantly higher in the immediate support group compared to the usual support group (20.7% [41/198] vs. 11.6% [23/199]; χ² = 5.486, *p* = 0.019) (Table 3).

**Table 3:** Quit rates at one year (Intention-to-Treat)

Group	Usual Care (*n* = 199)	Immediate Support (*n* = 198)	*p*
Quitters	23 (11.6%)	41 (20.7%)	0.019
Non-quitters	176 (86.4%)	157 (79.2%)	

## Discussion

This multicenter randomized trial found that immediate appointment scheduling for smoking cessation clinics substantially improved 12-month abstinence rates among patients with chronic airway diseases. The quit rate in the intervention group was 20.7% versus 11.6% in the usual care group. In addition, participants in the immediate support arm were more likely to attempt quitting, access pharmacological treatment, and maintain longer treatment durations. These findings demonstrate that a simple, pragmatic intervention embedded in pulmonary outpatient settings can produce meaningful, long-term gains in smoking cessation outcomes.

Despite diagnosis and well-documented health risks, a substantial proportion of patients with CAD continue smoking [5, 6]. This pattern reflects a complex interplay of behavioral, psychological, and structural factors, including high nicotine dependence, psychiatric comorbidities such as depression and anxiety, and socioeconomic barriers [7, 8]. Our findings reinforce the urgent need for proactive and accessible cessation interventions tailored to the real-life barriers encountered by this patient population.

This 12-month analysis builds on a previous phase of the same multicenter randomized trial, which demonstrated that immediate scheduling of smoking cessation clinic (SCC) appointments significantly improved short-term access to support and quit rates at 1 week and 3 months [10, 11]. The present findings confirm that these early gains are not transient but translate into significantly higher sustained abstinence at 12 months. Importantly, this effect was achieved in a real-world setting, using a simple, scalable intervention embedded in routine outpatient care.

In line with prior literature, our results support the value of structured interventions that establish direct linkage to cessation services, thereby improving patient engagement, adherence to pharmacotherapy, and ultimately, quit success [12]. Immediate appointment scheduling likely mitigates key behavioral barriers, such as procrastination, low perceived readiness, or difficulty navigating access pathways that often deter people who smoke from independently initiating cessation attempts [13]. Furthermore, the higher utilization of pharmacological therapies and longer treatment durations observed in the immediate support arm suggest that systematic clinical follow-up promotes more consistent and effective use of available cessation tools.

Notably, although a higher number of patients in the intervention group relapsed, this should be interpreted in light of the greater proportion of individuals who attempted to quit in this group. Thus, when evaluated proportionally, the higher relapse count likely reflects increased engagement with cessation efforts rather than diminished intervention effectiveness. This pattern aligns with the well-established relapsing nature of tobacco dependence, where multiple quit attempts are often required before sustained abstinence is achieved [14]. These findings highlight the importance of ongoing behavioral and pharmacological support to sustain abstinence and reduce relapse risk.

The results of this study align with a growing body of randomized controlled trials and meta-analyses supporting high-intensity smoking cessation programs for high-risk populations. High-intensity interventions are generally defined as programs that include multiple counseling sessions, sustained patient engagement, and the use of pharmacotherapy. Such interventions have demonstrated enhanced effectiveness, particularly in patients with COPD, resulting in higher quit rates and reduced exacerbation frequency [15, 16]. While the intervention in our study does not fully meet al.l criteria for a high-intensity program, immediate referral to smoking cessation services represents a key initial step by facilitating early access and engagement at a teachable moment. Embedding this low-cost, high-impact strategy into routine clinical workflows may enhance the reach and effectiveness of more comprehensive cessation interventions across respiratory care settings.

This study has several limitations. Although smoking status was biochemically verified using exhaled carbon monoxide in a subset of patients, abstinence was primarily based on self-report due to feasibility constraints in routine clinical practice. The study was conducted in secondary and tertiary care centers in Türkiye; while this may limit generalizability to primary care or other healthcare systems, it also reflects real-world clinical practice across different levels of care. The use of an alternating allocation sequence instead of true randomization with allocation concealment introduces a potential risk of selection bias, although baseline characteristics were comparable between groups. Importantly, loss to follow-up was not random. Participants lost to follow-up were more likely to have COPD and higher baseline nicotine dependence, both of which are associated with lower smoking cessation success. As these individuals were classified as people who smoke in the intention-to-treat analysis, this likely resulted in a conservative estimate of the intervention effect, suggesting that the true effectiveness of the intervention may be greater than observed. In addition, the study did not include a formal economic evaluation. Future research should assess the cost-effectiveness and implementation feasibility of immediate referral strategies across diverse healthcare settings.

In conclusion, immediate scheduling of appointments for smoking cessation services substantially improves one-year quit rates in patients with chronic airway diseases. Healthcare providers and systems should consider implementing streamlined, proactive referral processes as a core component of comprehensive CAD management. Doing so could close a longstanding treatment gap and help reduce tobacco-related morbidity and mortality in this high-risk population.

## Data Availability

The data that support the findings of this study are available from the corresponding author upon reasonable request.
